# Combined AKT and MEK Pathway Blockade in Pre-Clinical Models of Enzalutamide-Resistant Prostate Cancer

**DOI:** 10.1371/journal.pone.0152861

**Published:** 2016-04-05

**Authors:** Paul Toren, Soojin Kim, Fraser Johnson, Amina Zoubeidi

**Affiliations:** The Vancouver Prostate Centre, Department of Urologic Sciences, Faculty of Medicine, University of British Columbia, Vancouver, BC, Canada; Innsbruck Medical University, AUSTRIA

## Abstract

Despite recent improvements in patient outcomes using newer androgen receptor (AR) pathway inhibitors, treatment resistance in castrate resistant prostate cancer (CRPC) continues to remain a clinical problem. Co-targeting alternate resistance pathways are of significant interest to treat CRPC and delay the onset of resistance. Both the AKT and MEK signaling pathways become activated as prostate cancer develops resistance to AR-targeted therapies. This pre-clinical study explores co-targeting these pathways in AR-positive prostate cancer models. Using various *in vitro* models of prostate cancer disease states including androgen dependent (LNCaP), CRPC (V16D and 22RV1) and ENZ-resistant prostate cancer (MR49C and MR49F), we evaluate the relevance of targeting both AKT and MEK pathways. Our data reveal that AKT inhibition induces apoptosis and inhibits cell growth in PTEN null cell lines independently of their sensitivity to hormone therapy; however, AKT inhibition had no effect on the PTEN positive 22RV1 cell line. Interestingly, we found that MEK inhibition had greater effect on 22RV1 cells compared to LNCaP, V16D or ENZ-resistant cells MR49C and MR49F cells. *In vitro*, combination AKT and MEK blockade had evidence of synergy observed in some cell lines and assays, but this was not consistent across all results. *In vivo*, the combination of AKT and MEK inhibition resulted in more consistent tumor growth inhibition of MR49F xenografts and longer disease specific survival compared to AKT inhibitor monotherapy. As in our *in vitro* study, 22RV1 xenografts were more resistant to AKT inhibition while they were more sensitive to MEK inhibition. Our results suggest that targeting AKT and MEK in combination may be a valuable strategy in prostate cancer when both pathways are activated and further support the importance of characterizing the dominant oncogenic pathway in each patient’s tumor in order to select optimal therapy.

## Introduction

Medical or surgical castration remains the first line of systemic therapy for metastatic prostate cancer (PCa) since its discovery over 70 years ago [1]. Unfortunately, cure remains elusive following castration and patients inevitably progress to develop castrate resistant prostate cancer (CRPC). Potent androgen receptor (AR) pathway inhibitors such as enzalutamide (ENZ) and abiraterone are now commonly used in the treatment of patients with CRPC. While survival is improved, resistance nonetheless inevitably develops to these agents [2]. It is anticipated that with the increased clinical use of these more potent AR pathway inhibitors that targeting approaches against non-AR driven resistance pathways will gain increasing importance [3]. Therefore, understanding and targeting pathways implicated in resistance has important clinical relevance.

The PI3K/AKT/mTOR and RAF/MEK/ERK signaling pathways play an important role in cell survival, treatment resistance, and cooperate to facilitate PCa progression to CRPC [4–8]. Both AKT [9, 10] and ERK [11, 12] signaling pathways are up-regulated with CRPC and are associated with poor outcome [13, 14]. There is extensive cross-talk between these two pathways as well as with other oncogenic pathways [15, 16]. We have previously showed that both AKT and ERK are activated following treatment with ENZ in PCa cells [17]. Targeting AKT alone is not sufficient to induce conditional lethality due the feedback signaling leading to activation of AR and therefore targeting AKT alone is not a good strategy to combat ENZ resistance [18]. Interestingly, dual inhibition of PI3K/AKT and MEK/ERK pathways has shown promise in pre-clinical models of other cancers [19–22]. Results of the combination of an mTOR inhibitor with a MEK inhibitor in the transgenic *NKx3*.*1-PTEN* murine prostate cancer model further supports the rationale for a combined approach in prostate cancer [14] therapy.

Therefore, we set to investigate combination AKT plus MEK inhibitor therapy in human prostate cancer models, particularly ENZ-resistant prostate cancer models. We selected a panel of cell lines including ENZ-resistant LNCaP-derived cell lines as well as the 22RV1 cell line. The 22RV1 prostate cancer cell line possesses activation of the MEK/ERK pathway [23], while the ENZ-resistant MR49C and MR49F are recognized to be more dependent on the AKT pathway [24]. We demonstrate that combination blockade of the AKT and MEK does improve responses compared to monotherapy in some of ourin *in vitro* and *in vivo* prostate cancer experiments. Notably, the results vary considerably between model systems with the absence of additional benefit in some cases, highlighting the need to appropriately identify which patients will benefit most from a combination approach.

## Materials and Methods

### Prostate cancer cell lines

The human prostate cancer LNCaP and 22RV1 cell lines used in this study were kindly provided by Dr. Leland W.K. Chung[25] (1992, MDACC, Houston Tx). V16D (castrate resistant), MR49F and MR49C cells (enzalutamide resistant) were derived through serial xenograft passage of LNCaP cells as previously described [26] (S1 Fig). Briefly, LNCaP cells were injected to mice and when the serum PSA reached 50ng/ml mice were castrated. When the serum PSA again reached 50ng/ml 5–6 weeks later, tumors were called castrate resistant. V16D cells were derived from an LNCaP xenograft resistant to castration. Tumors were excised and cell lines were generated in androgen-free media. For MR49C and MR49F cell lines, when LNCaP xenografts reached the castrate resistant state, they were treated with 10mg/Kg of ENZ with a subsequent PSA decline to low serum values. When the serum PSA returned to castrate resistant levels or higher, tumors were called ENZ-resistant. These tumors were then passed 3 times in castrate mice treated with ENZ and cell lines were generated. Cells were maintained in RPMI 1640 medium (Invitrogen) supplemented with 10% fetal bovine serum (FBS) at 37°C in 5% CO2 atmosphere, with 10μM ENZ added to all media for MR49C and MR49F cells.

### Reagents

The AKT inhibitor AZD5363 was provided by AstraZeneca (Macclesfield, UK). ENZ was purchased from Shanghai Haoyuan Chemexpress (Shanghai, China), PD0235901 from Selleck Chem (Houston, TX) and LY294002 and UO126 from Sigma-Aldrich (St Louis, MO). LY294002 is a reversible PI3K inhibitor; AZD5363 is a competitive pan-AKT inhibitor [27]. PD0325901 is a competitive MEK1/2 inhibitor while UO126 inhibits MEK1/2 in a non-competitive, selective manner [28]. Stock solutions of AZD5363, PD0235091, UO126 and LY294002 were prepared in dimethyl sulfoxide (DMSO, Sigma-Aldrich); ENZ was prepared in H_2_O.

### Cell proliferation assays

Cell viability was assessed in 96-well culture plates using the WST-1 reagent and/or crystal violet assay, as described previously[26].

### Cell cycle analysis

Cell cycle analysis with propidium iodide staining was performed as previously described [29]. Relative DNA content was analyzed by FACS Canto II flow cytometer using the cyflogic v1.2.1 software (www.cyflogic.com) for analysis.

### Caspase-3 activity assay

Caspase-3 activity was assessed using the Caspase 3 Assay kit, with the acetyl Asp-Glu-Val-Asp 7-amido-4-methylcoumarin (Ac-DEVD-AMC) fluorometric substrate (Enzo Scientific). Thirty micrograms of whole cell lysate was incubated with caspase-3 substrate AC-DEVD-AMC at 37.5°C for 2.5h and caspase-3 activity was quantified with a fluorometer with excitation set at 365nm and emission 460nm. Fold change differences from control were calculated following subtraction of readings from blank wells without lysate.

### Western blot analysis

Total proteins were extracted in RIPA buffer as previously described [29]. 30–50 µg of protein lysate was separated by SDS-PAGE, and western blot was performed using primary antibodies PSA, AR (Santa Cruz Biotechnology), vinculin (Sigma-Aldrich, St. Louis, MO), PARP, p-AKT Ser473, AKT, p-ERK, total ERK, total S6 and p-S6 (Cell Signaling Technology, Danvers, MA). Detection of secondary antibodies was performed using the ODYSSEY IR imaging system (Li-COR Biosciences) or ECL (Amersham Biosciences, Piscataway, NJ, USA).

### Quantitative reverse transcription (RT)-PCR

RNA extraction and RT-PCR were performed as previously described [30]. Real time monitoring of PCR amplification of cDNA was performed using the following primer pairs and probes: *AR* (Hs00171172_m1), *PSA* (Hs00426859_g1), and *GAPDH* (Hs03929097_g1) (Applied Biosystems, Foster City, CA) on the ABI PRISM 7900 HT Sequence Detection System (Applied Biosystems) using TaqMan Gene Expression Master Mix (Applied Biosystems). Target gene expression was normalized to *GAPDH* levels in respective samples as an internal control.

### Animal treatment

Six week old male castrated athymic nude mice (Harlan Sprague-Dawley, Inc.) were injected subcutaneously with 2x10^6^ MR49F cells (suspended in 0.1ml Matrigel; BD Biosciences) on both flanks. Nine mice per arm were randomized to vehicle (0.1% methylcellulose), AZD5363 100mg/kg BID, PD0325901 5mg/kg OD or the combination. ENZ 10mg/kg was administered prior to inoculation and continued until tumors reached 200mm^3^. Tumor measurements were measured biweekly and tumor volume calculated using the formula l x w x d x 0.5236. Drugs were administered as an oral gavage 5 days on, 2 off. PSA levels were measured weekly using automated enzymatic immunoassay (Cobas, Montreal, Quebec, Canada). Mice were sacrificed if the total tumor burden was >2000mm^3^ or had >20% loss of body weight. For the 22RV1 xenografts, 2x10^6^ cells were inoculated into the left flank of nude castrate mice. Eight mice per arm were randomized to vehicle, AZD5363 100mg/kg BID, selumitinib (AZD6244; ARRY-142886) 25mg/kg BID, and the combination. The 22RV1 xenografts were sacrificed when tumor volume was >1500mm^3^. All mice were monitored regularly for their clinical condition under the supervision of a veterinarian at the University of British Columbia. At sacrifice, all mice were deeply anesthetized with isoflurane prior to being euthanized with CO2. Tumors collected at sacrifice were divided into parts and snap frozen in liquid nitrogen or fixed in formalin. All animal procedures were performed according to Canadian Council on Animal Care guidelines and with approval of the Animal Care Committee of the University of British Columbia (protocol # A12-0210).

### Immunohistochemistry

A tissue microarray of all MR49F xenograft tumors was constructed using a manual tissue microarrayer (Beecher Instruments, Inc., Sun Prairie, WI). Immunohistochemical staining was performed as previously reported [31]. All comparisons of staining intensities were done at 20X magnification on triplicate samples by a pathologist blinded to treatment assignment.

### Statistical analysis

All results are expressed as the mean ±SEM, with one-way ANOVA used to detect significant differences between multiple treatments. When ANOVA showed significant differences (p<0.05), Tukey’s HSD post-hoc test was used to compare means. Tumor growth velocity was calculated using linear regression. Kaplan Meier survival analysis compared cancer specific survival (CSS) and overall survival, with the log-rank test used to compare groups. CSS was defined as time from treatment start until total tumor volume exceeded 2000mm^3^ and overall survival as the time from treatment start until sacrifice. The combination index (CI) was calculated using the Calcusyn software (Biosoft, Cambridge, UK). A CI <1 indicates synergy, a CI >1 indicates antagonistic interactions.

## Results

### Effect of targeting MEK and AKT pathways on AR signaling pathway

**Fig 1:**
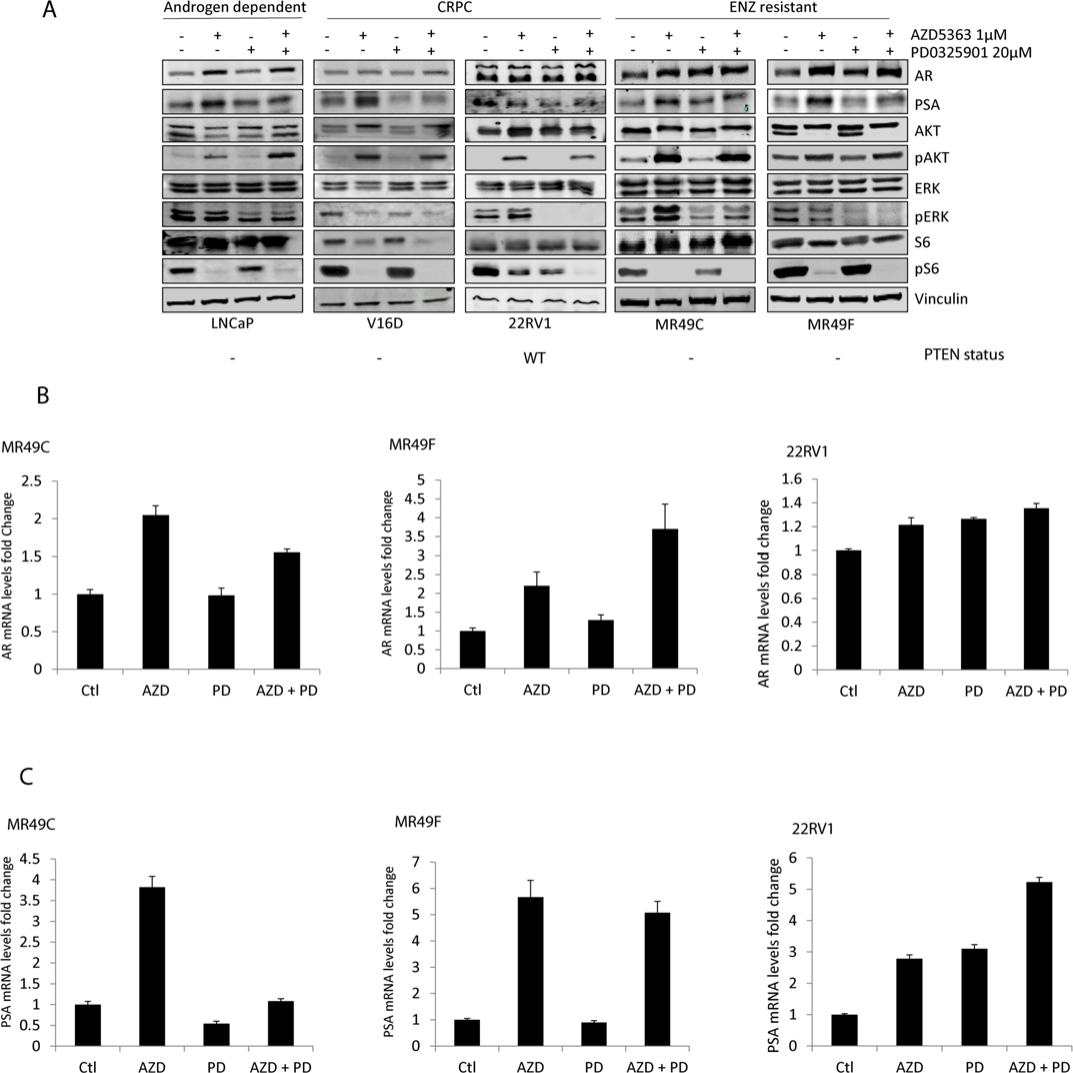
Effect of AKT and MEK inhibition on downstream signaling pathways an AR signaling pathway. **(A)** Effect of AKT and MEK inhibition on downstream signaling pathways. Androgen dependent PCa cell line LNCaP, CRPC (V16D and 22RV) and ENZ resistant cell lines MR49C, MR49F cell lines were treated with AZD5363 1μM, PD0325901 20 μM alone or in combination for 48 hours. Total proteins were extracted and western blots were performed using AR, PSA and PI3K/AKT pathway signalling proteins as indicated. Representative blots of duplicate experiments are shown. **(B-C)** Effect of AKT and MEK inhibition on AR pathway. MR49C, MR49F and 22RV cell lines were treated with AZD5363 1μM, PD0325901 20 μM alone or in combination for 48 hours. RNA was extracted from different cell lines and quantitative real time were performed using Taqman probes for AR **(B)** and AR target gene PSA **(C)**. Representative results of biologic duplicates with technical triplicates are shown.

To investigate the relevance of AKT and MEK pathways in PCa, we targeted respective pathways using AZD5363 (AKT inhibitor) and PD0325901 (MEK inhibitor). We evaluated their effects alone and in combination in models of different stages of prostate cancer including androgen sensitive cells (LNCaP), castrate resistant (V16D, 22RV1) and ENZ-resistant cells (MR49C and MR49F) (Fig 1). Blocking AKT with AZD5363 was evaluated by its effect on AKT downstream effector p6SK and not on AKT phosphorylation itself because of the nature of AZD5363. Basically, AZD5363 induces increased AKT phosphorylation which is inactive, a phenomenon occurs with many ATP competitive, catalytic inhibitors of AKT, and is due to the protein being held in a hyperphosphorylated but catalytically inactive form as a consequence of compound binding as has been established previously. AZD5363 was found to induce a decrease in S6K phosphorylation in all cell lines; this effect was more pronounced in the PTEN null cells MR49C, MR49F, LNCaP and V16D cells compared to PTEN positive 22RV1 cells (Fig 1). Similar effects were observed using other MEK inhibitor (UO126) and AKT inhibitor (LY294006) or in combination with Ay (Fig 1, S2 Fig). In 22RV1 cells, PD325901 completely abrogates ERK phosphorylation while AZD5363 had no effect on AKT downstream effector S6K phosphorylation which was only with combination of AZD5363 and PD0325901 (Fig 1). However, no additional decrease in pERK levels was observed with the combination compared to MEK inhibitor monotherapy. Moreover, we observed that AKT inhibition increased both AR and PSA at protein and RNA levels in LNCaP and its derivatives (Fig 1). Although AZD5363 increased AR and PSA levels in all cell lines, the effect of PD0325901 on AR and PSA levels alone or in combination with AZD5363 varied between cell lines (Fig 1). In contrast to LNCaP-based cell lines, PSA was further increased by the combination of AZD5363 and PD0325901 in 22RV1 cells. Notably, these results mirrored similar data with the previously tested successful combination of AZD5363 and ENZ where the changes in AR expression also differed between 22RV1 and LNCaP-based cell lines (S2 Fig) [32]. Taken together, we conclude that AKT and MEK cooperate to regulate the AR pathway.

### Effect of combination of MEK and AKT blockade on cell apoptosis and proliferation

**Fig 2:**
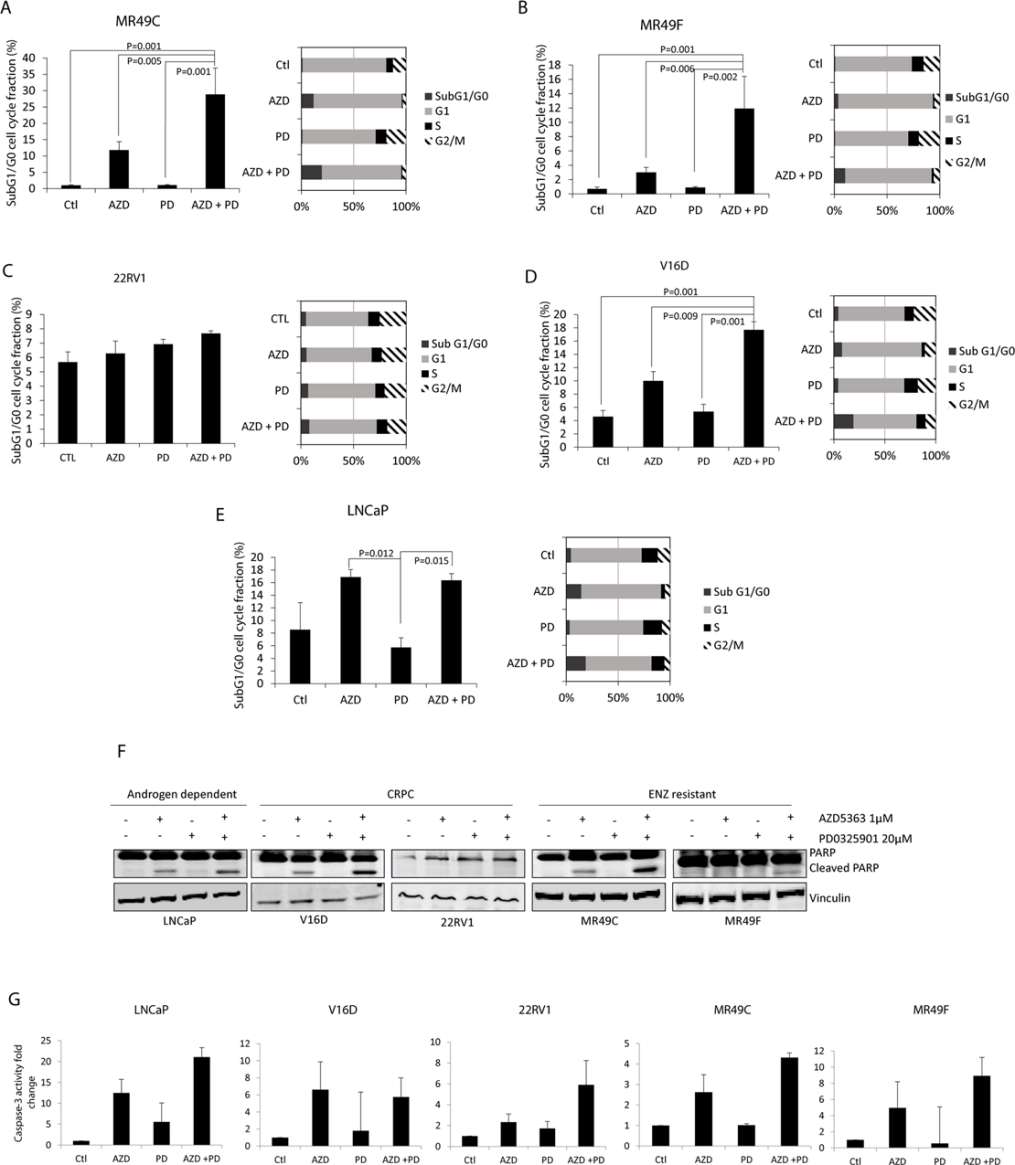
Combination AZD5363 + PD325901 increases apoptosis. **(A-E)** Propidium iodide flow cytometry cell cycle analysis of indicated cell lines shows increased apoptotic cell cycle fraction (SubG1/G0) (left panels). Means of triplicate experiments are plotted +/- SEM. Representative results of all cell cycle populations are shown in right panels. **(F)** Indicated cells were treated with AZD5363 1μM, PD0325901 20μM, or the combination for 48 hours. Proteins were extracted and western blot was performed using PARP antibody, vinculin was used as a loading control. Representative blots of two or more experiments are shown. **(G)** Caspase-3 activity in MR49C, MR49F, 22RV1, LNCaP and V16D cell lines treated with AZD5363 and/or PD0325901 for 24 hours. Mean fold change +/-SEM of pooled values from at least two biologic duplicates are shown.

To determine the biological activity of targeting AKT and MEK signaling pathways on cell apoptosis, we treated our panel of cell lines with AZD5363, PD0325901 or the combination. Our data showed that combination of targeting AKT and MEK induces apoptosis as shown by increased sub G0/G1 cell cycle population. This effect was greater than AZD5363 monotherapy in CRPC cells V16D (17% vs 10%, P = 0.009) and ENZ-resistant MR49C (29% vs 12%, p = 0.005) and MR49F (12% vs 3%, p = 0.006) cells. None of the treatments exhibited any significant changes in the sub G1/G0 fraction in 22RV1 cells (Fig 2). In contrast, in androgen-sensitive LNCaP cells, combination treatment therapy did not show further induction of sub G1/G0 compared to AZD5363 monotherapy (17% vs 16%, p = 0.76). S-phase and G2/M fractions appeared to decrease to a similar amount in MR49C, MR49F and V16D cells with AZD5363 or the combination, with significant differences for both treatments from control(P<0.001). Moreover, AZD5363 and PD325901 combination treatment induced cleaved PARP in LNCaP-based cell lines compared to PTEN positive cells 22RV1 cells (Fig 2). While not statistically different, similar trends were observed with caspase 3 activity assays (Fig 2).

**Fig 3:**
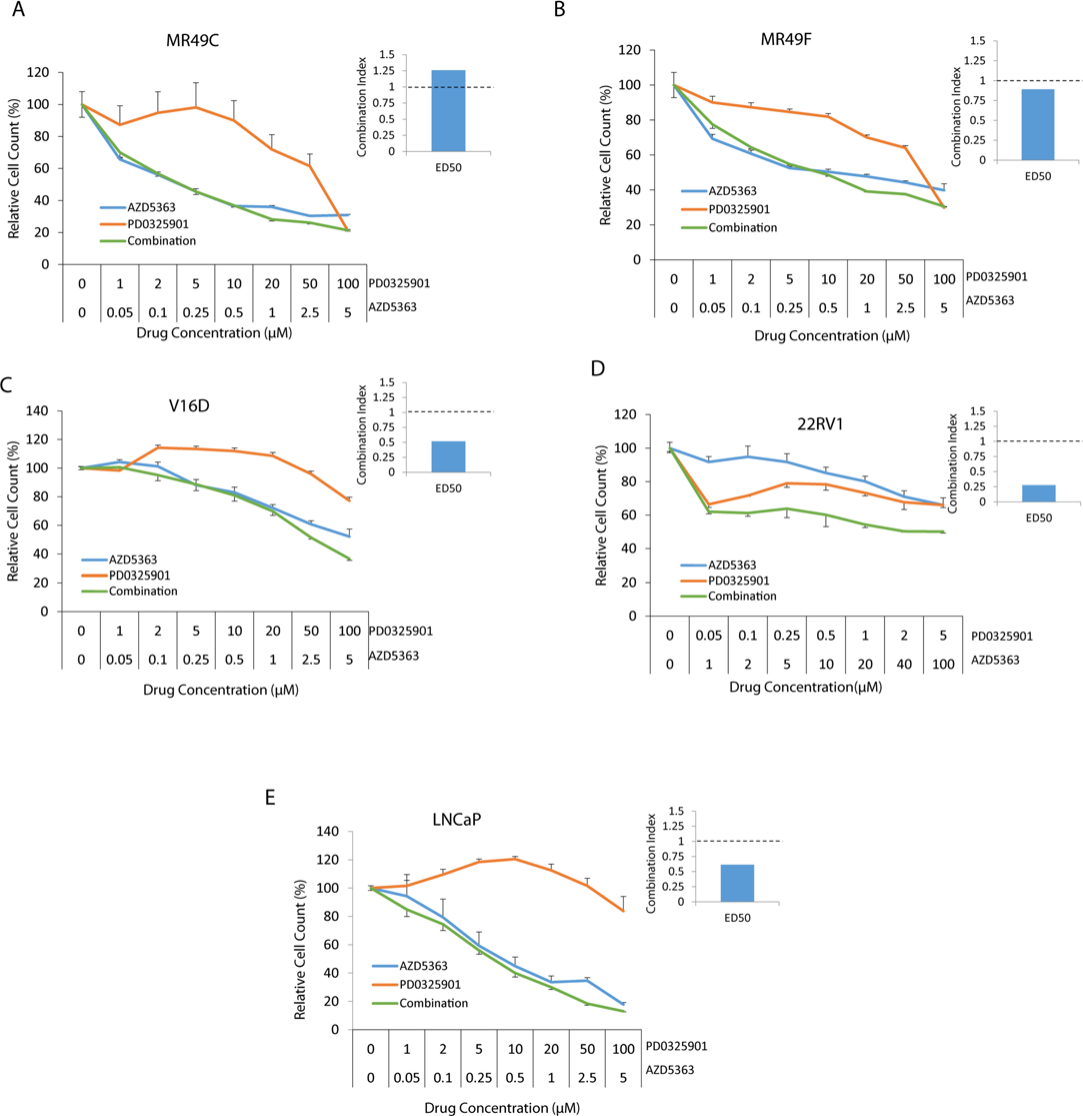
Effect of AZD5363 and PD0325901 on cell viability. **(A-E)** MR49C, MR49F, 22RV1, LNCaP and V16D cell lines were treated with AZD5363 and/or PD0325901 as indicated for 48 hours and cell viability was assessed using WST-1 assay. Pooled results of biologic triplicates with technical triplicates are shown. Combination indices are shown inset, with values <1 indicating synergy.

We next investigated if the effect observed in cell apoptosis can be translated to cell viability. Our data show that targeting AKT using AZD5363 affects cell viability in all cell lines tested (Fig 3) while targeting MEK using either PD0325901 (Fig 3) or UO126 (S3 Fig) had greater activity in 22RV1 cells compared to the other cell lines further confirming our data on cell cycle population and PARP cleavage. Although we observed trends for decreased viability and synergy with the combination compared to monotherapy in LNCaP cells and V16D cells, in MR49C and MR49F cells the combination did not appear synergistic (Fig 3). This was further confirmed using the combination of MEK inhibitor U0126 with the AKT inhibitor LY294006 in MR49C, MR49F cells (S3 Fig). Synergy was observed in 22RV1 cells with a combination index <1 (Fig 3, S3 Fig).

### Targeting AKT and MEK pathways in combination inhibit tumor growth and improved cancer specific survival

Since the activity of combination therapy showed differences related to PTEN expression, we investigated *in vivo* how targeting AKT and MEK will influence tumor growth in two possible clinical settings (PTEN positive and negative tumors). We used ENZ-resistant MR49F cells and CRPC 22RV1 cells to assess the *in vivo* efficacy of the combination of targeting AKT and MEK pathways.

**Fig 4:**
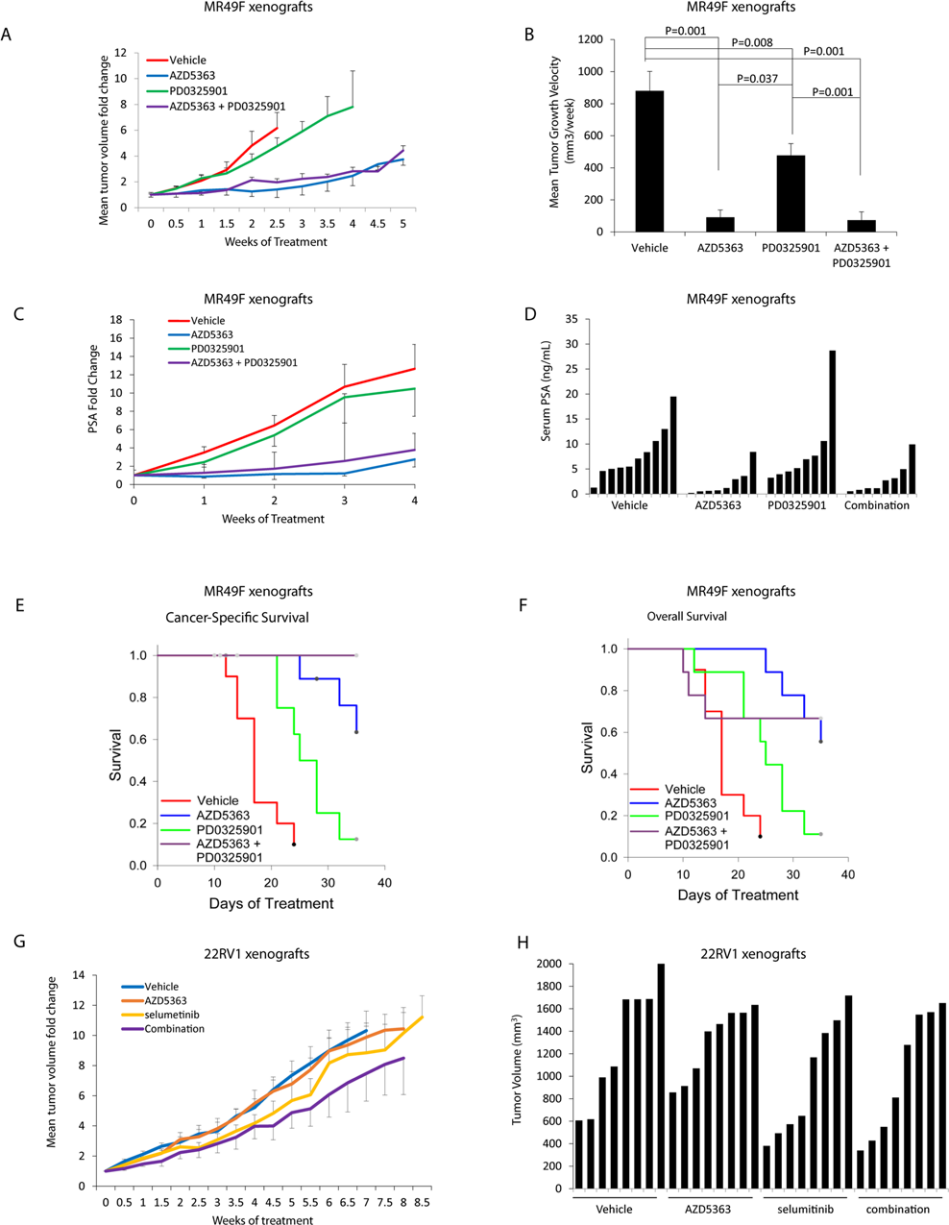
Effect of targeting MEK and AKT using PD0325901 and AZD5363 *in vivo*. After establishment of tumors (200mm3) from subcutaneous injection of ENZ resistant MR49F cells in castrated mice under the pressure of 10mg/kg daily of ENZ, mice were treated with 100mg/kg, AZD5363 100mg/kg BID, PD0325901 5mg/kg QD or AZD5363 100mg/kg BID + PD0325901 5mg/kg QD. **(A)** Representative data of MR49F mean tumor volume over 4 weeks is shown. **(B)** Mean MR49F tumor growth velocity for each treatment group +/-SEM calculated using linear regression estimation of tumor growth velocity for each mouse. **(C**) Mean MR49F weekly PSA plotted as fold change from baseline for each treatment group +/-SEM. **(D)** Waterfall plot of individual PSA measurements after 3 weeks of treatment for all MR49F xenografts in the study. **(E-F)** Kaplan-Meier cancer specific survival and overall survival curves for treatment arms of MR49F xenografts. **(G)** Mean 22RV1 xenograft volume following treatment with vehicle, AZD5363 100mg/kg BID, selumetinib 25mg/kg QD or AZD5363 100mg/kg BID + selumetinib 25mg/kg QD. Mean fold change in tumor size is plotted +/-SEM. **(H)** Waterfall plot of 22RV1 xenograft tumor volumes after 6 weeks of treatment.

The MR49F xenografts were sensitive to AZD5363, making assessment of additional benefit with MEK blockade challenging. Monotherapy treatment with PD0325901 demonstrated tumor growth inhibition and demonstrated a non-significant trend to lower serum PSA compared to vehicle (Fig 4). Combination treatment with AZD5363 and PD0325901 in the MR49F xenografts did not result in greater decreases in tumor volume compared to AZD5363 monotherapy, but the combination did have significantly improved cancer specific survival (CSS) (P<0.001) compared to vehicle- and PD0325901- treatment arms (Fig 4). Median CSS was 17 days for vehicle-treated mice, 25 days for PD0325901-treated mice, and was not reached for either AZD5363 alone or in combination with PD0325901. No mice in the combination arm were euthanized due to tumor size after 35 days of treatment. Serum PSA was significantly reduced in both the AZD5363 and the combination treatment arms compared to vehicle (Fig 4).

We next assessed the combination of AKT and MEK pathway inhibition using 22RV1 xenografts in castrated mice. AZD5363 was again used as an AKT inhibitor while Selumetinib was chosen as a MEK inhibitor which may be more clinically relevant as it is currently in clinical evaluation and was recently approved for treatment of melanoma. In contrast to the MR49F model, 22RV1 tumors were relatively insensitive to AZD5363, but did demonstrate tumor growth inhibition to both Selumetinib and the combination of Selumetinib plus AZD5363. With all 22RV1 tumors growing relatively robustly despite treatment, no significant differences were found between treatment arms, though the greatest tumor growth inhibition was seen with combination treatment (Fig 4).

**Fig 5:**
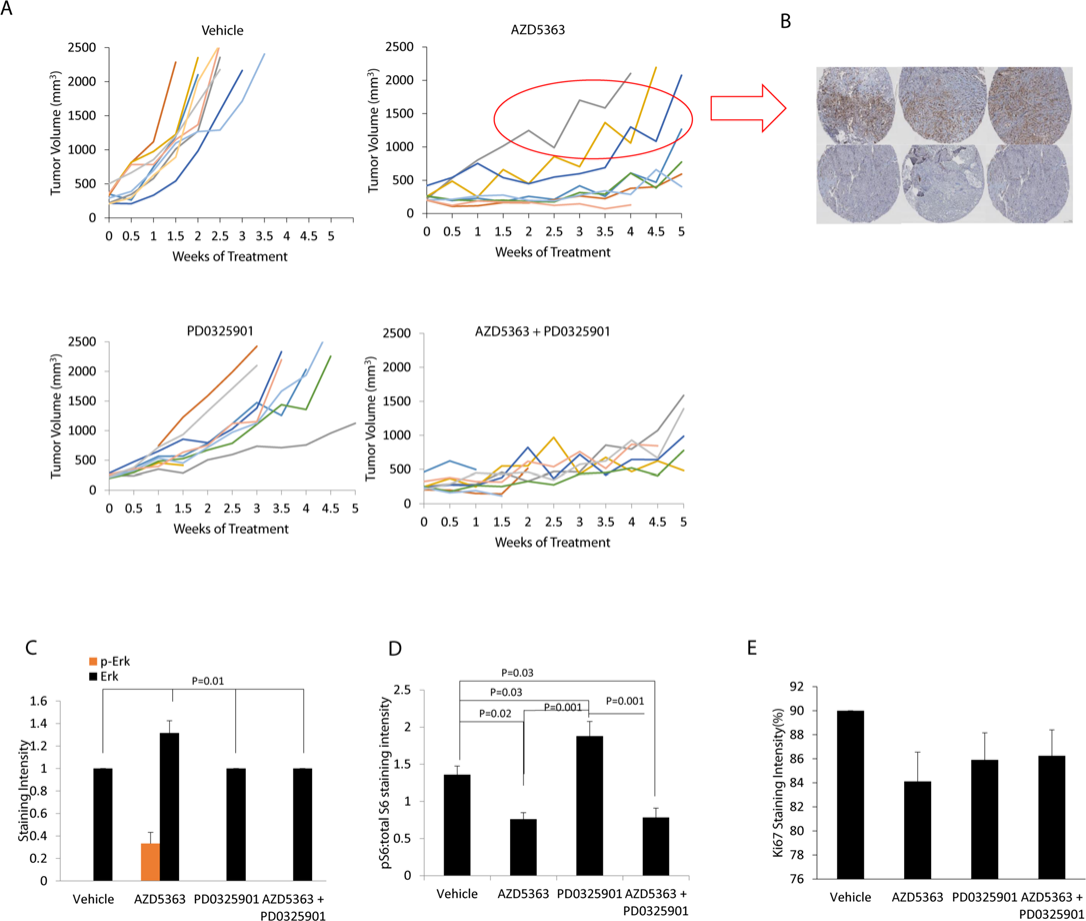
Analysis of tumors treated with combination MEK + Akt inhibition. **(A)** Individual tumor growth curves for all mice in the study grouped by treatment. **(B)** Immunohistochemistry of pERK demonstrates detectable levels in 3 mice with early resistance to AZD5363 treatment (top); 3 other mice treated with AZD5363 are shown for comparison (bottom). **(C)** Staining of microarray specimens demonstrates that positive pERK staining was only evident in these mice. **(D)** The proportion of pS6 is decreased with AZD5363 and to a greater extent with combination AZD5363 + PD0325901. **(E)** Ki67 staining as a marker of proliferation. A tissue microarray was constructed using tumor samples in each group (7–9 per group). Mean scores of staining intensity graded by a blinded pathologist are shown +/- SEM.

Analysis of the individual mice tumor volumes in the MR49F model suggests that combined blockade may benefit a subset of mice (Fig 5). In the AZD5363 monotherapy arm, while most tumors responded well, in select cases, the tumor growth inhibition was minimal. However, in the combination therapy group, no outliers were noted (Fig 5). Notably, immunohistochemistry staining of the MR49F tumors demonstrated pERK staining in the mice which demonstrated resistance to AZD5363 (Fig 5). No significant pERK staining was seen in the mice who responded to the treatment (Fig 5) nor were differences in pS6 staining or Ki67 staining detected between combination treatment and the AZD5363 monotherapy arm (Fig 5).

## Discussion

Despite the success of castration and newer potent AR pathway inhibitors such as enzalutamide, treatment resistance occurs in all cases. Persistent AR signaling is usually clinically evident by a rising PSA. The persistence of AR-signaling may be driven by several mechanisms of resistance including AR splice variants, intra-tumoral production of androgens and activation of alternate pathways which support AR signalling, including the AKT and MEK pathways [33]. Combination therapy in PCa holds potential for increasing patient survival by blocking these resistance pathways.

Early clinical experience in PCa patients suggests that targeting AKT alone is not an effective strategy. Clinical trials of AKT inhibitor monotherapy in CRPC have demonstrated limited success with this approach [34, 35]. Prior *in vitro* work suggests that PI3K/AKT inhibition can result in up-regulation of the Raf/MEK/ERK pathway [36], and our results in the MR49F xenograft model also suggests this may occur in some cases. The well-recognized crosstalk between these pathways provides a rationale for combining both of these inhibitors [19–22, 37–39]. Clinical experience with dual targeting of MEK and AKT pathways is limited; early results in advanced malignancies suggests that dual targeting of both pathways improves oncologic efficacy at the cost of greater toxicity[40].

Our study highlights the differences which can occur between AR positive PCa models and especially the relevance of PTEN status when targeting AKT and MEK pathways. Interestingly, targeting the AKT pathway increased PSA transcription in the surviving cells and this effect, noted previously by others [41], may be important to consider in the design of future clinical trials with these targeted agents since PSA is commonly used as a marker of progression. In the LNCaP-derived ENZ-resistant models, MEK inhibition added a modest benefit compared to AKT inhibition; interestingly the synergy appeared greater *in vivo* than *in vitro*. In 22RV1 cells, MEK inhibition alone had significant impact on cell proliferation and signaling, but the combination of MEK and AKT inhibition demonstrated minimal additional benefit *in vitro*. Thus, while improvements in anticancer activity were noted by some metrics with the combination in each model, each model appeared relative addicted to AKT and MEK, respectively, thus limiting the benefit of combination therapy.

Combined targeting of the MEK and AKT pathway has been investigated in several other cancers in pre-clinical models, with results supporting the use of the combination [19–22, 37–39]. In a pre-clinical prostate cancer *in vivo* study, PD0325901 was assessed in combination with the mTOR inhibitor rapamycin [14]. While we did not observe any significant decrease in Ki67 with the combination, this may be related to collecting all of our tumors at the end of study at which point the xenograft tumors were treatment-resistant. Recently, Park et al, showed the combination of selumitinib and the pan-PI3K inhibitor GSK2126458 improved tumor growth inhibition compared to either monotherapy in AR-negative DU145 and PC3 xenografts[42]. Overall, their results in AR-negative models are comparable to our results. Taken together, our data suggest that the efficacy of targeting AKT and MEK is independent of the disease state of prostate cancer and that the efficacy correlates with the activation of these pathways in the tumor.

Our study is limited by several aspects of our prostate cancer models. LNCaP cells appear to be highly dependent on the AKT pathway. Nonetheless, as AR positive, PSA-producing cell lines with an activated PI3K/AKT pathway, they do resemble a common CPRC phenotype encountered [43]. Moreover, our ENZ-resistant models demonstrate several phenotypes consistent with clinical ENZ-resistant disease, such as persistent AR nuclear localization, AR F876L mutation, and upregulation of steroiodogenic enzymes [26, 32, 44]. 22RV1 cells harbor androgen splice variants, which predict a poorer response to AR-pathway inhibitors [45]. The presence of the enzalutamide-agonistic F876L mutation and the presence of dominant AR splice variants limited our ability to test for the efficacy of combined AKT and MEK inhibition together with AR inhibition in advanced prostate cancer models. Further, while our in vitro studies were not performed in androgen deficient media, it is unclear whether this would result in significantly different results, particularly as CRPC tumors can produce their own androgens through *de novo* synthesis pathways [46]. Accordingly, our results in castrate conditions *in vivo* were not substantially different.

In conclusion, our results in ENZ-resistant CRPC cell models suggest that combination treatment with AKT and MEK inhibition may be a rational combination in a subset of resistant prostate cancer cases needing further investigation. Our results also suggest the importance of targeting inhibitors to tumor pathways which are known to be activated; in well-selected patients monotherapy may be as effective as combination therapy. Overall, this emphasizes the need for rational, biomarker-driven selection of patients as targeted therapeutics are evaluated in clinical trials.

## Supporting Information

S1 Fig MR49C cells are resistant to ENZ. 5X10^4^cells were seeded in 12-well plates, then treated with different concentrations of ENZ (0, 0.1, 0.3, 1, 3, 10uM). After 72 hours exposure, culture medium changed fresh media alone (Recover) or with ENZ (continue). 48Hrs later, cell viability was analyzed with crystal violet assay.
(TIF)

S2 Fig Effect of combination AKT plus MEK inhibition with alternate inhibitors on cell signalling. **(A)** MR49C and MR49F cell lines were treated with LY294006 20μM, UO126 10 μM, or combination for 48 hours. Total protein was extracted and wester blots were performed using AR, PSA and PI3K/AKT pathway signalling proteins. **B)** MR49C and MR49F cell lines were treated with LY294006 20μM, UO126 10 μM, or combination for 24 hours. RNA was extracted and quantitative real time PCR results of Taqman probes for AR.
(TIF)

S3 Fig Effect of combination AKT plus MEK inhibition with alternate inhibitors on cell proliferation. **(A)** Indicated cell lines were treated with LY294002 and UO126 at indicated doses and cell viability was assessed using crystal violet. Results shown are pooled values of triplicate repeats of biologic triplicate experiments +/- SEM. **(B)** Combination indices calculated for AZD5363 + PD0325901 combination (left) and UO126 + LY294002(right) from pooled crystal violet proliferation results. Values <1 indicate synergy.
(TIF)
