# Supplementary figures and images for: Combined AKT and MEK Pathway Blockade in Pre-Clinical Models of Enzalutamide-Resistant Prostate Cancer
Source: PLoS One. 2016 Apr 5;11(4):e0152861. doi: 10.1371/journal.pone.0152861 (PMC4821639; doi:10.1371/journal.pone.0152861)

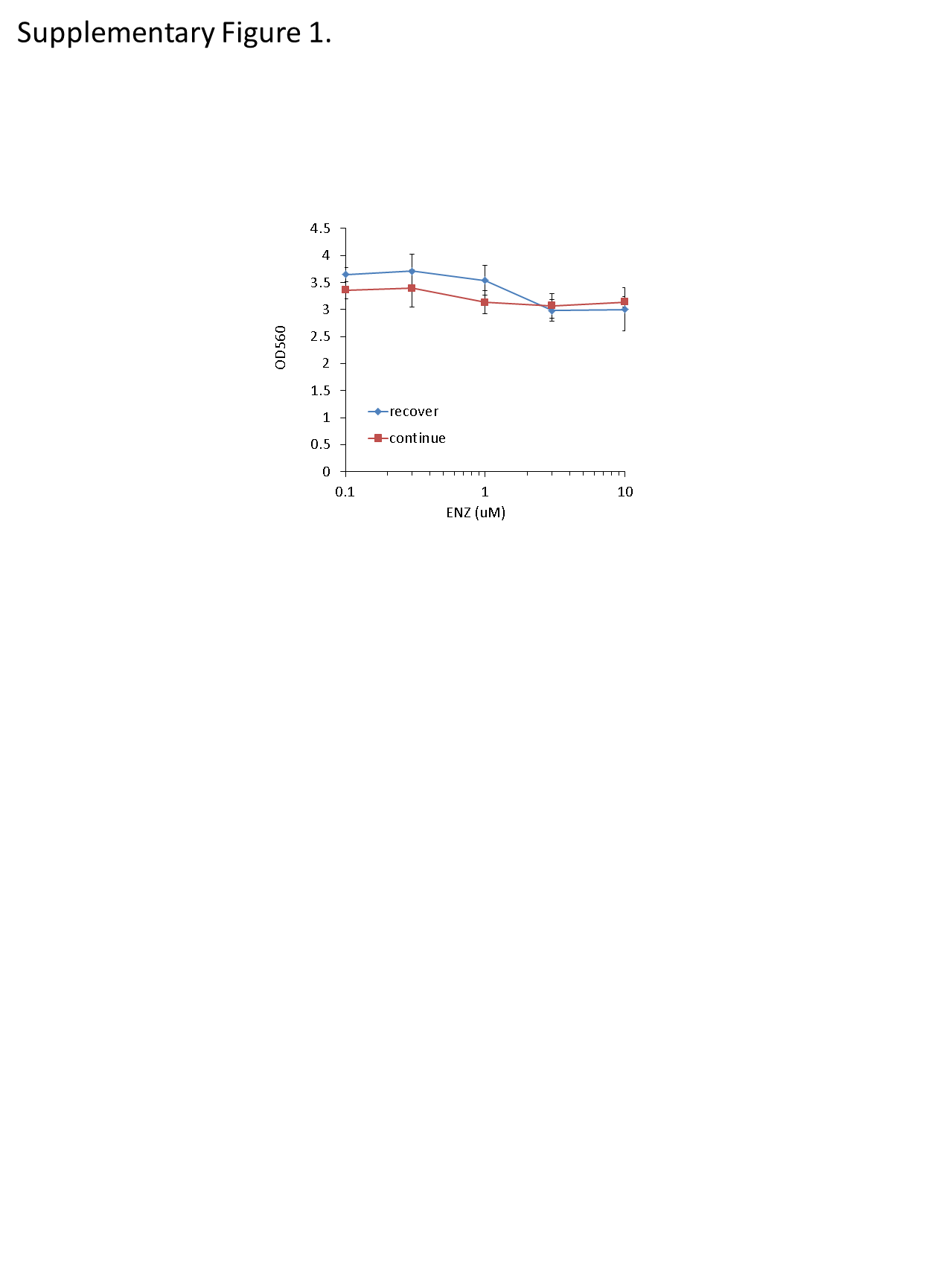

Supplement: S1 Fig — 5X104cells were seeded in 12-well plates, then treated with different concentrations of ENZ (0, 0.1, 0.3, 1, 3, 10uM). After 72 hours exposure, culture medium changed fresh media alone (Recover) or with ENZ (continue). 48Hrs later, cell viability was analyzed with crystal violet assay. (TIF) [file pone.0152861.s001.TIF]

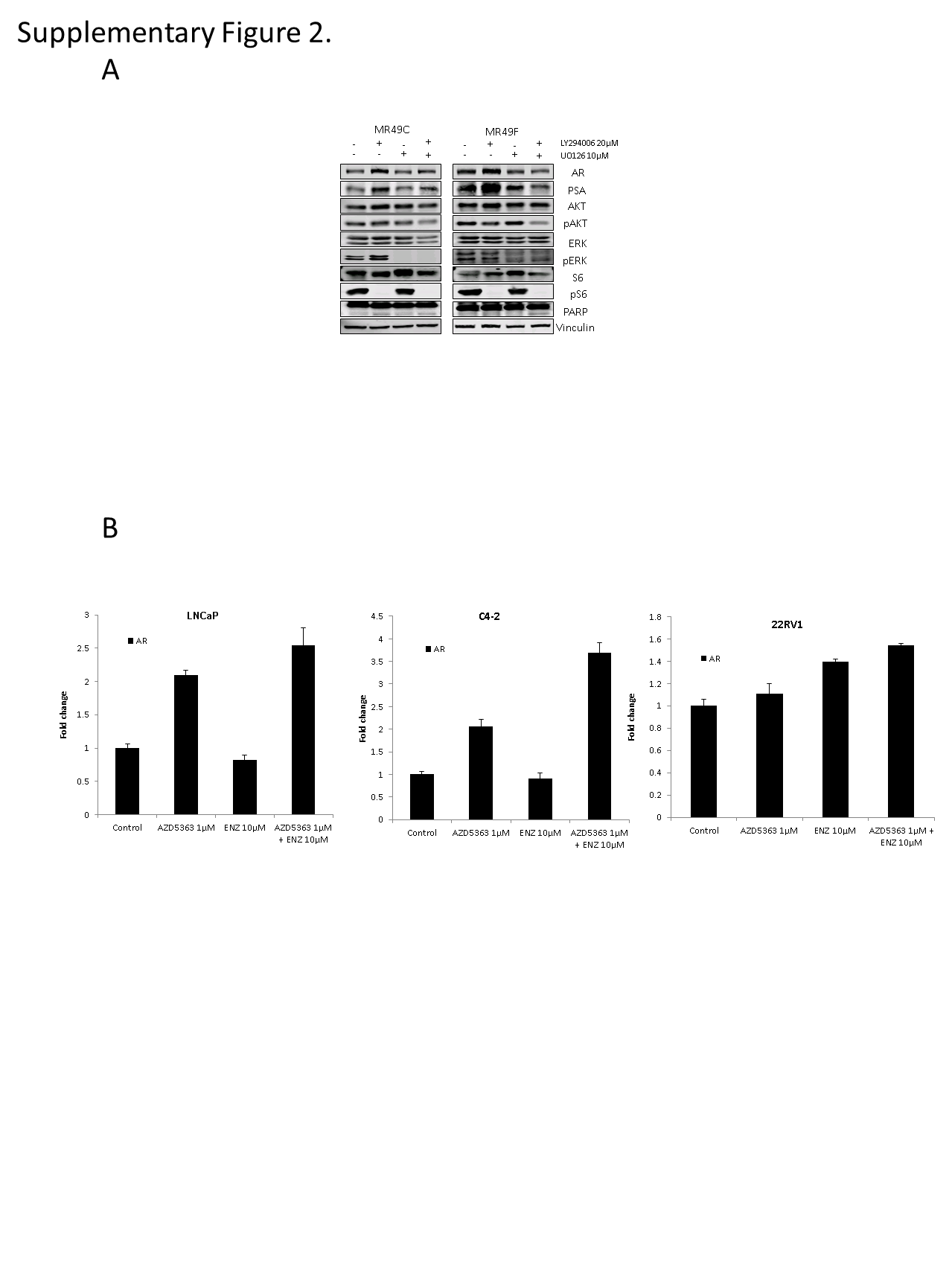

Supplement: S2 Fig — (A) MR49C and MR49F cell lines were treated with LY294006 20μM, UO126 10 μM, or combination for 48 hours. Total protein was extracted and wester blots were performed using AR, PSA and PI3K/AKT pathway signalling proteins. B) MR49C and MR49F cell lines were treated with LY294006 20μM, UO126 10 μM, or combination for 24 hours. RNA was extracted and quantitative real time PCR results of Taqman probes for AR. (TIF) [file pone.0152861.s002.TIF]

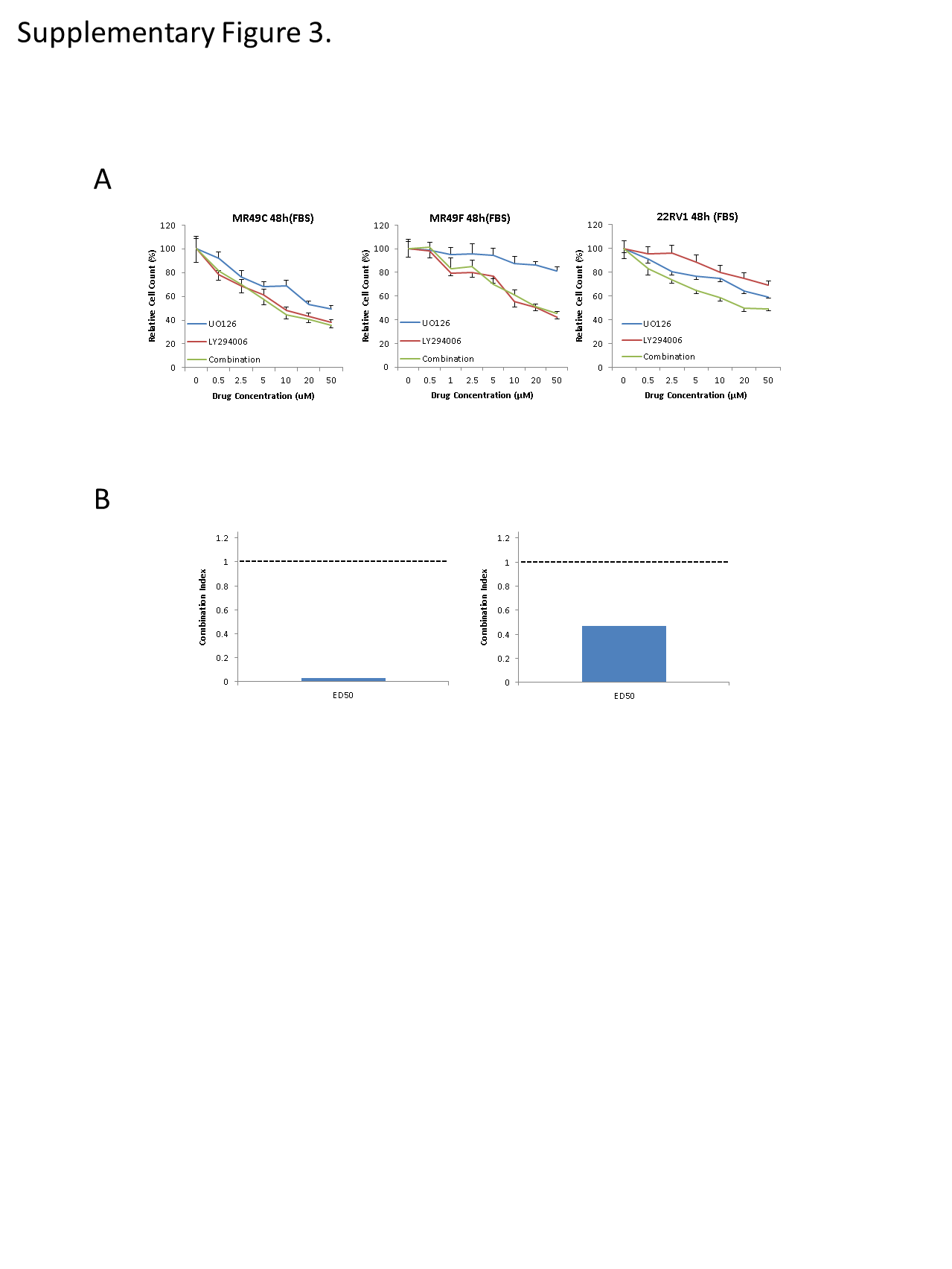

Supplement: S3 Fig — (A) Indicated cell lines were treated with LY294002 and UO126 at indicated doses and cell viability was assessed using crystal violet. Results shown are pooled values of triplicate repeats of biologic triplicate experiments +/- SEM. (B) Combination indices calculated for AZD5363 + PD0325901 combination (left) and UO126 + LY294002(right) from pooled crystal violet proliferation results. Values <1 indicate synergy. (TIF) [file pone.0152861.s003.TIF]
